# Correction: Global cellular response to chemotherapy-induced apoptosis

**DOI:** 10.7554/eLife.01816

**Published:** 2013-11-07

**Authors:** Arun P Wiita, Etay Ziv, Paul J Wiita, Anatoly Urisman, Olivier Julien, Alma L Burlingame, Jonathan S Weissman, James A Wells

Wiita AP, Ziv E, Wiita PJ, Urisman A, Julien O, Burlingame AL, Weissman JS, Wells JA. 2013. Global cellular response to chemotherapy-induced apoptosis. *eLife* **2**:e01236. doi: 10.7554/eLife.01236. Published 29 October 2013

In Figure 3—figure supplement 3, the underlining had been incorrectly shifted five characters to the left.

The article has now been corrected.

